# A human iPSC-based neural spheroid platform for modelling glioblastoma infiltration using high-content imaging

**DOI:** 10.1038/s41598-025-30914-5

**Published:** 2025-12-13

**Authors:** Victoria S. K. Tsang, Federica Riccio, Aimee S. Wilson, Hannah Nudds, Jason D. Coombes, Heiko Wurdak, Harry J. C. J. Bulstrode, Ivo Lieberam, Davide Danovi

**Affiliations:** 1 Centre for Gene Therapy and Regenerative Medicine, King’s College London, London, UK; 2 Department of Basic & Clinical Neuroscience and Institute of Psychiatry, King’s College London, London, UK; 3 Centre for Developmental Neurobiology and MRC Centre for Neurodevelopmental Disorders, King’s College London, London, UK; 4 Wellcome MRC Cambridge Stem Cell Institute, University of Cambridge, Cambridge, UK; 5 Department of Neurosurgery, Addenbrooke’s Hospital, Cambridge, UK; 6 School of Medicine, University of Leeds, Leeds, UK; 7 Translational Medicine and Therapeutics, Queen Mary University of London, London, UK; 8 Department of Clinical and Movement Neurosciences, University College London, London, UK; 9 Saint Louis University, St Louis, USA; 10 Migration Biotherapeutics, Cardiff, UK

**Keywords:** Glioblastoma, Cancer migration, Induced-pluripotent stem cells, Neural spheroid, High-content imaging, Stem cell modelling, Cancer, Cell biology, Neuroscience

## Abstract

Glioblastoma is the most aggressive adult brain tumour, characterised by resistance to therapy and high recurrence due to diffuse infiltration. We developed a physiologically relevant co-culture model, combining patient-derived glioblastoma cell lines with cortical-like neural spheroids differentiated from human induced pluripotent stem cells. Using high-content imaging, we demonstrate that GBM20 and GBM1 cell lines migrate directionally along axons toward neural spheroids in live imaging assays and infiltrate spheroids extensively in endpoint assays, unlike non-cancerous neural stem cells. A proof-of-principle drug screen identified PF 573228 (FAK inhibitor) and motixafortide (CXCR4 inhibitor) as potent suppressors of GBM20 and GBM1 infiltration, respectively. Bulk RNA sequencing revealed gene expression profiles correlating with invasive behaviour and drug sensitivity. This platform offers a valuable model for studying glioblastoma infiltration along axons and provides proof-of-principle that migration can serve as a measurable and actionable phenotype to screen therapeutic vulnerabilities in glioblastoma.

**Supplementary Information:**

The online version contains supplementary material available at 10.1038/s41598-025-30914-5.

## Introduction

Glioblastoma (GB) is the most common and aggressive form of primary brain tumour in adults, with a median overall survival of just 12 to 15 months following diagnosis and standard-of-care treatment with temozolomide^1–8^. GB is defined by its IDH1/IDH2-wildtype status and classified as a World Health Organization (WHO) grade IV tumour, reflecting its high malignancy^3,5^. A major barrier to effective treatment is the pronounced inter- and intra-tumoural heterogeneity in genetic mutations and cellular phenotypes, which complicates therapeutic targeting and drives drug resistance^9–11^. Moreover, the highly infiltrative nature of GB precludes complete surgical resection and temozolomide treatment, promoting tumour recurrence^6,12,13^.

A key contributor to the aggressive invasiveness of GB is a subpopulation of neoplastic cells called the GB stem-like cells (GSCs)^14–17^. GSCs exhibit hallmark features of stemness, including self-renewal and multipotency, and are found at the tumour core and in infiltrative margins, where they play a key role in tumour recurrence and therapy resistance^17–20^. Most importantly, GSCs are highly invasive and use conserved mechanisms of invasion, emphasising their critical role in disease progression. Their invasive capacity is mediated by intrinsic processes, including cytoskeletal remodelling and upregulation of surface receptors that regulate adhesion and motility^15^. These mechanisms facilitate GSC migration along two primary routes, the vascular endothelium and axonal tracts within the white matter of the brain, although the mechanisms underlying the route selection remain unclear^14,15,21^.

An increased understanding of the prognostic significance of tumour invasion has driven the development of advanced *in vitro* models that more accurately recapitulate GB complexity for drug screening purposes^22^. Among these, patient-derived GSC lines have emerged as a valuable research tool. These lines faithfully preserve the stem cell-like properties, genetic landscape, and transcriptional profile of the parental tumour, even after extended passaging, thereby maintaining key features of the *in vivo* tumour phenotype^23,24^. Particularly, patient-specific GSCs have been used to generate physiologically relevant model systems, including glioma spheroids and GB cerebral organoids that better represent the complexity of GB and the tumour microenvironment^25^.

High-content imaging combines automated microscopy with quantitative single-cell analysis, allowing detailed, large-scale assessment of cellular phenotypes^26,27^. In oncology research, high-content imaging enables dynamic studies of cell behaviours such as migration, especially when using label-free modalities like phase contrast to avoid phototoxicity during long-term live-cell assays^28,29^. We and others have made use of these advancements, alongside computational and multi-omics approaches, to enhance drug screening and target discovery in complex tumour models like GB^30–33^.

In this study, we present a novel *in vitro* co-culture platform combining patient-derived GB cells with axons projecting from human iPSC-derived neural spheroids. This system recapitulates tumour-neuron interactions within a physiologically relevant microenvironment, allowing mechanistic investigation of GB cell migration and infiltration that drive tumour recurrence. Using high-content imaging, we reveal significant differences in the migration patterns of two different patient-derived GB cell lines and provide insights into the mechanisms underlying GB cell line-specific infiltration strategies, with potential implications for anti-infiltrative therapies. Additionally, we correlate migration phenotypes with drug responses identified through a screening of relevant inhibitors. This drug screen provides proof-of-principle that migration is a measurable phenotype with potential for guiding precision therapies against GB infiltration.

## Results

### Characterisation of iPSC-derived cortical neural spheroid

**Fig. 1:**
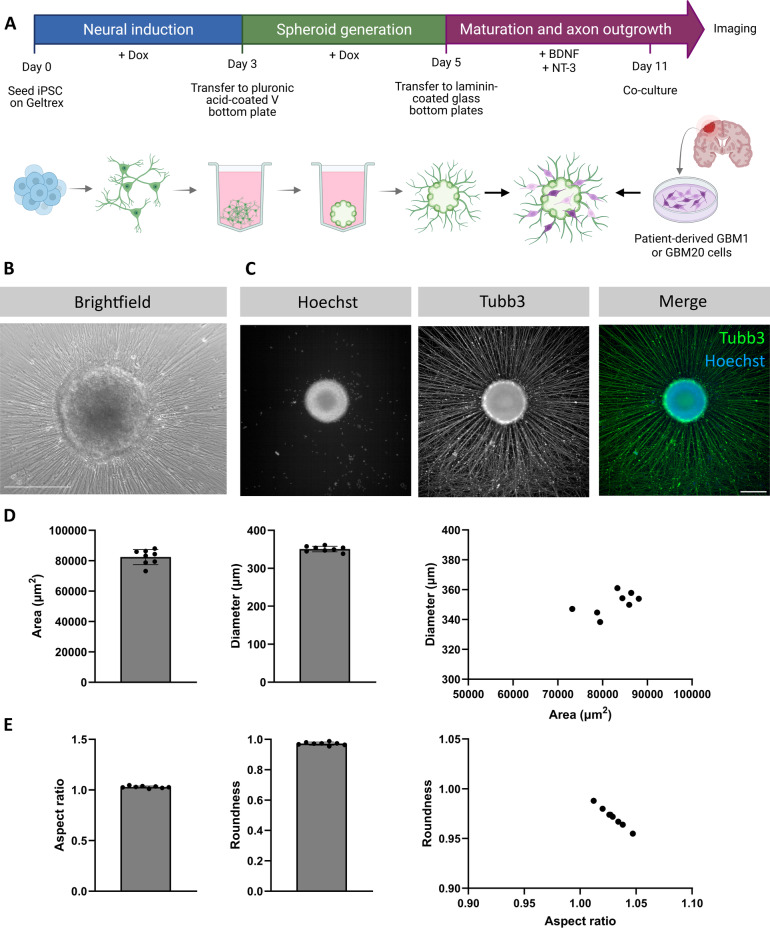
Co-culture of human iPSC-derived neural spheroids and patient-derived GB cells. (**A**) Timeline and schematic diagram of the transcription-factor mediated neural induction from Fernandopulle et al., neural spheroid generation using Pluronic acid-coated V-bottom plates, maturation, axon outgrowth, and co-culture with patient-derived cells. Created in BioRender, https://BioRender.com/gr57vkk (**B**) Representative brightfield image captured on the × 10 microscope of a neural spheroid generated through forward reprogramming. Scale bar = 200 μm. (**C**) Representative confocal images of the neural spheroid and axons (Hoechst in blue and Tubb3 in green). Scale bar = 200 μm. (**D**) Morphometric characterisation of neural spheroids, including area, diameter, roundness, and aspect ratio. Data are presented as individual values with mean ± SD from eight independent experiments (n = 8).

To generate an *in vitro* model suitable for the characterisation of infiltration of GB cells and amenable to drug-screening studies, we combined the culture of patient-derived GB cell lines with human iPSC-derived neural spheroids with radially extending axons. Wildtype human iPSCs are differentiated into excitatory cortical-like neurons using NGN2-mediated forward programming differentiation^34^. Following the induction phase, early neuronal progenitors were placed into non-adherent Pluronic acid-coated V-bottom plates where they self-aggregated into individual spheroids of reproducible size and shape (Fig. 1A) . Then, spheroids were transferred onto laminin-coated plates where they adhered to the culture substrate and extended long, radially organised neurites (Fig. 1B). Immunofluorescence staining performed on the spheroids revealed positive expression of the axonal marker βIII-tubulin (Tubb3) in the projecting neurites (Fig. 1C), validating the generation of a neuronal substrate suitable for GB co-culture assays.

To ensure reproducibility of the neural spheroids, we quantified area, diameter, roundness, and aspect ratio across eight independent experiments. Quantitative analysis confirmed high morphological consistency between spheroids. The average spheroid area was 82,000 ± 5,000 µm^2^ (mean ± SD; CV = 6.0%), corresponding to a mean diameter of 351 ± 7 µm (CV = 2.1%) (Fig. 1D). Roundness was 0.97 ± 0.01 (CV = 1.0%) and aspect ratio 1.03 ± 0.01 (CV = 1.0%) (Fig. 1E). Roundness and aspect ratio values close to 1 indicate that the spheroids were perfect spheres. These results demonstrate robust and reproducible spheroid formation, supporting their suitability for screening applications.

### Patient-derived GB cells migrate along axons toward the neural spheroid *in vitro*

Having established a co-culture of neural spheroids with emerging axons, we next examined the cell behaviour of two different patient-derived GB cell lines, GBM1 and GBM20, in this axon-rich microenvironment. GBM1, first described by Wurdak et al., was derived from a primary, treatment-naïve tumour^9,35^, while GBM20, reported by Polson et al., was obtained from a recurrent tumour previously treated with chemoradiotherapy and the IMA950 peptide vaccine^35^. The human neural stem cell line NS17, with features of self-renewal and multipotency^36^, was examined in parallel as a non-tumourigenic control^36^.

**Fig. 2:**
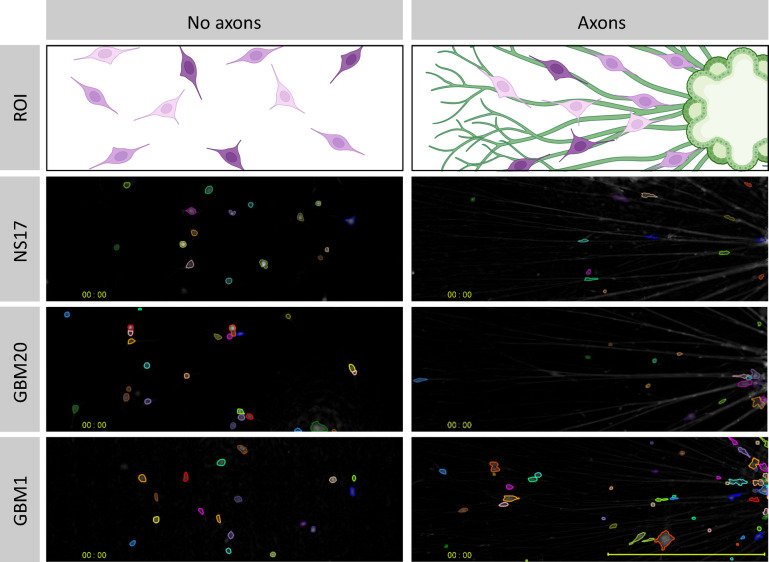
Live imaging of neural spheroids co-cultured with NS17, GBM20, and GBM1 cells. Schematic representation of the experimental setup showing cells cultured either in the presence of neural spheroids with extending axons or alone on laminin-coated plates. Phase-contrast images acquired using the Livecyte depict NS17, GBM20, and GBM1 cells at the start of the time-lapse assay (00:00) with cell mask overlays. Scale bar = 500 μm. No axons: cells plated alone in laminin-coated wells. Axons: cells co-cultured with neural spheroids with extending axons.

**Fig. 3:**
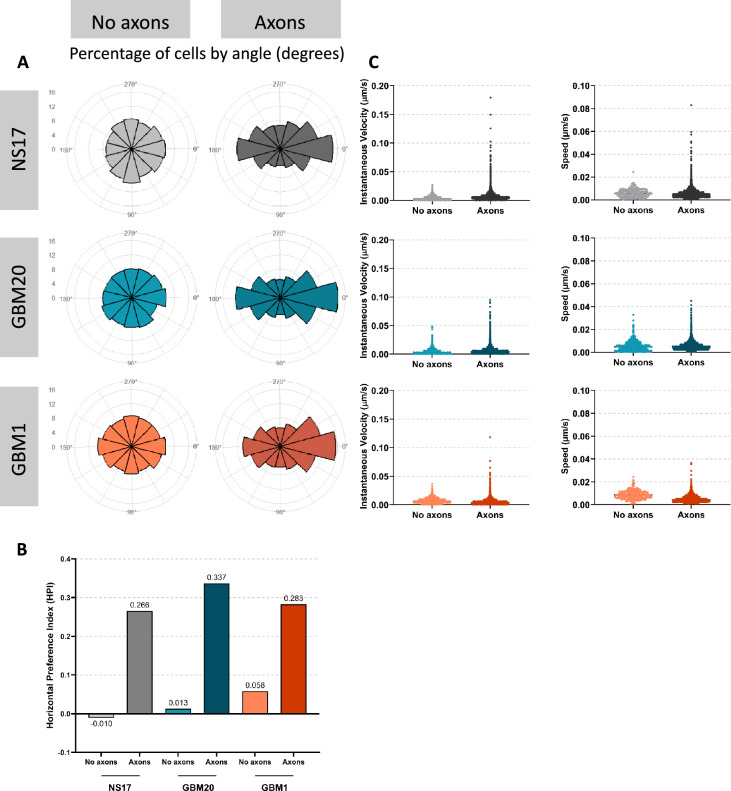
Motility analysis of live imaging assays of neural spheroids co-cultured with NS17, GBM20, and GBM1 cells. (**A**) Polar histograms summarise the percentage of cells migrating by angle in degrees. The data represent an independent experiment (n = 1) with 1000 cells analysed, divided into angle bins. (**B**) Quantification of the Horizontal Preference Index (HPI), where a positive result indicates a horizontal bias. (**C**) Quantification of the instantaneous velocity and speed of the NS17, GBM20, and GBM1 cells with or without axons.

The GB and NS17 cell lines were cultured either in the presence of neural spheroids with extending axons or alone on laminin-coated plates. Visualisation of live-cell imaging videos showed that all three cell lines aligned with and migrated along neuronal axons (Fig. 2 and Supplementary Videos). To quantify this cell-axon interaction, single-cell tracking data (~ 1,000 cells per condition) from a representative experiment were analysed, providing robust cell-level quantification while acknowledging the limitation of relying on a single experimental replicate. To determine whether the direction of cell migration along axons was random or biased toward the neural spheroid, we measured the directionality of individual migration tracks with polar histograms (Fig. 3A). To quantify any directional migration, we calculated a Horizontal Preference Index (HPI), where a positive value indicates bias for movement along the horizontal axis (Fig. 3B). In the absence of axons, migration was isotropic; NS17 and GBM20 showed no significant horizontal preference, with an HPI value near zero (-0.010 and + 0.013, respectively) and by the uniform distribution of their polar histograms. In contrast, the presence of axons induced a powerful horizontal bias (HPI =  + 0.266 for NS17 with axons; + 0.337 for GBM20 with axons; + 0.283 for GBM1 with axons). The HPI findings were statistically significant (*p* < 0.0001), confirming a contact-guided migration along the horizontal axons. Also, all three co-culture conditions showed a clear enrichment of cell movement toward the neural spheroid positioned on the right side of the imaging field, with 10% of NS17 control cells and 12% of GBM20 and GBM1 cells migrating at an angle of 0° (Fig. 3A).

Notably, all three cell lines indicated an increase in instantaneous velocity when cultured on axons compared with axon-free conditions (Fig. 3C). A notable change in mean migration speed was detected only for GBM1 cells on axons, with no corresponding effect in the other cell lines. These findings suggest that axons function as a permissive, orienting substrate that enhances guidance cues, particularly for GBM1 cells. Furthermore, the increased variability in directionality and velocity on axons underscores the heterogeneity of cell responses, supporting the need for further investigation into temporal dynamics and GB subtype-specific migration mechanisms.

### GB cells exhibit cell line-specific infiltration in neural spheroid co-culture

**Fig. 4:**
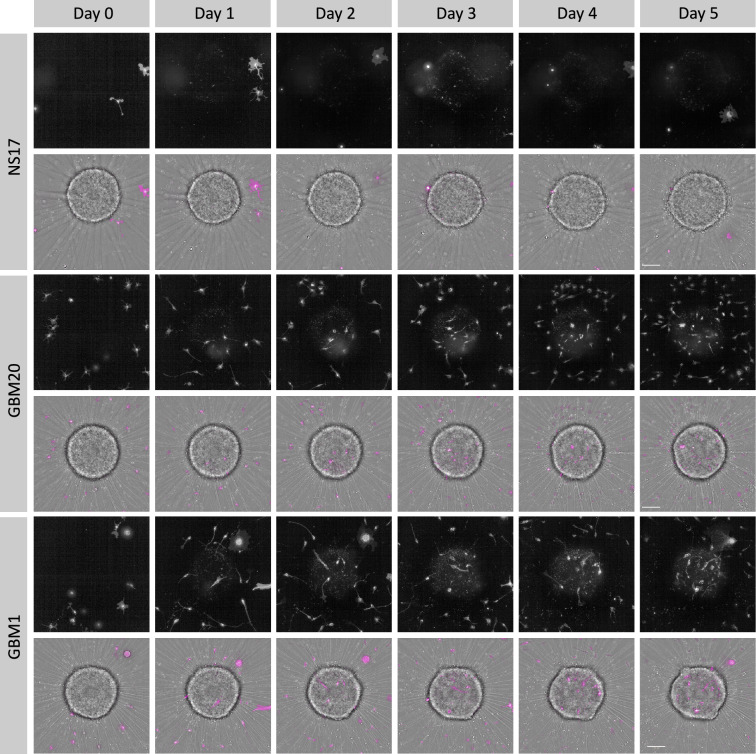
Endpoint imaging of neural spheroids co-cultured with NS17, GBM20, and GBM1 cells. Representative maximum projection confocal images acquired on the Operetta CLS, showing co-cultures of the neural spheroids with NS17, GBM20, and GBM1 cells at Days 0 to 5. Scale bars = 100 μm.

**Fig. 5:**
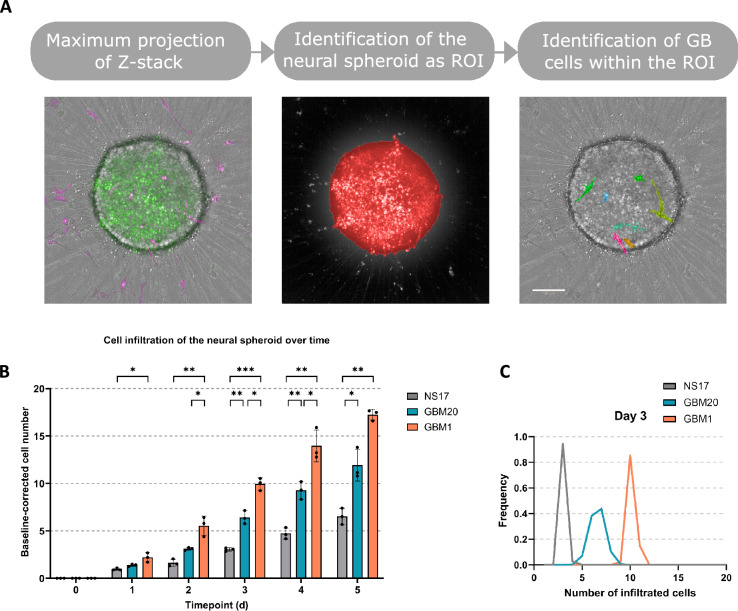
Infiltration analysis of endpoint imaging assays of neural spheroids co-cultured with NS17, GBM20, and GBM1 cells. (**A**) Analysis pipeline to quantify the GB cells within the neural spheroid from images acquired on the Operetta CLS and analysed using Harmony. Scale bars = 100 μm. (**B**) Integrated quantification of neural spheroid infiltration by NS17, GBM20, and GBM1 cells from Days 0 to 5. Data are baseline-corrected cell numbers and are presented as individual values with mean ± SD from three independent experiments (n = 3). Statistical significance was assessed using two-way ANOVA with Tukey’s multiple comparisons test performed on log₁₀(Y + 1)-transformed data (*, *p* < 0.05; **, *p* < 0.005; ***, *p* < 0.0005). (**C**) Normal distribution curves for NS17, GBM20, and GBM1 infiltration calculated from the means and standard deviation at Day 3. Z-factor = 0.701.

We hypothesised that cells migrating toward the neural spheroid would subsequently infiltrate it. To assess the infiltration potential of patient-derived GB cells relative to non-tumourigenic neural stem cells, NS17, GBM20, and GBM1 cells were labelled with a membrane-permeable cytoplasmic dye, seeded onto neural spheroids at an equal density of 840 cells per well, and imaged daily for five days (Fig. 4). A customised image analysis pipeline was later applied to endpoint images acquired from Days 0 to 5 to quantify the number of infiltrating cells across conditions (Fig. 5A).

All three cell lines exhibited a progressive increase in spheroid infiltration over five days (Fig. 5B). GBM1 showed the steepest increase, with a significant difference from NS17 observed as early as Day 1 (5.5 ± 1.0 vs. 1.6 ± 0.4 cells, *p* = 0.0219) and the greatest difference at Day 3 (9.9 ± 0.6 vs. 3.0 ± 0.2 cells, *p* = 0.0002). GBM20 also exhibited robust, though less pronounced, infiltration, reaching a significant difference from NS17 by Day 3 (6.4 ± 0.7 vs. 3.0 ± 0.2 cells, *p* = 0.0059). GBM20 infiltrated the neural spheroid more slowly than GBM1 (3.1 ± 0.2 vs. 5.5 ± 1.0 cells by Day 2, *p* = 0.0360). By Day 5, both GBM1 and GBM20 showed significantly higher infiltration than NS17, averaging 17.2 ± 0.6 (*p* = 0.0077) and 11.9 ± 1.6 (*p* = 0.0153) cells per spheroid, respectively, compared with 6.5 ± 0.9 cells for NS17. These consistent trends across replicates highlight the sustained invasive potential of GB-derived cells relative to non-tumorigenic controls, as well as the heterogeneity in infiltration dynamics between the two GB lines.

Analysis of infiltration value distributions further revealed a progressive divergence between patient-derived GB lines and the non-tumourigenic control (Fig. 5C). The Z-factor, a measure of assay quality that combines signal dynamic range and variability (values ≥ 0.5 indicate excellent robustness and separation between groups^37^) was used to assess assay performance. By Day 3, the distributions of GBM1 and GBM20 were clearly shifted relative to NS17, yielding a Z-factor of 0.701 and demonstrating strong discriminatory power over Days 4 and 5 (see also Figure S1A). These data identify Day 3 as the optimal time point for distinguishing differential infiltration phenotypes in this model, with potential utility for high-throughput anti-invasion compound screening.

### GB and neural spheroid co-cultures enable the identification of tumour-specific infiltration inhibitors

Having developed a quantitative pipeline to measure GB cell migration along axons and infiltration into the neural spheroid core, we next used this co-culture platform for a pilot drug screen. To investigate mechanisms regulating GB infiltration, a panel of 18 inhibitors (Table 1) targeting cytoskeletal dynamics, kinase signalling, and axonal guidance pathways that mediate glioma invasion was selected based on literature and clinical relevance^15,17,38^. This included established cytoskeletal disruptors such as inhibitors to actin polymerisation (latrunculin B), myosin II ATPase (blebbistatin), and ROCK1/2 (Y-27632), commonly used to reduce cell motility, along with targeted canonical GB signalling pathways involved in cell migration and invasion, including inhibitors to TGF-β receptor (SB 431542), PI3K/Akt (API-2), Protein Kinase C (PKC) (GF 109203X), and integrins (cilengitide), pathways often targeted in clinical evaluation^39–43^. Further targets included non-canonical regulators such as Hedgehog (HPI-1) and Wnt (IWP-2) pathways, and receptor tyrosine kinases, including FAK (PF 573228), FGFR (PD 173074), PDGFR (AC 710), and EGFR (iressa)^44–46^. Given the importance of axon-guided migration in GBM, we included inhibitors of Ephrin receptors (ALW-II-41–27 for EphA2, NVP-BHG712 for EphB4, and Ehp-inhibitor-1), and the CXCL12/CXCR4 axis (motixafortide), known to regulate directed migration in the hypoxic tumour core in immunotherapy^47,48^. Finally, we tested repurposed drugs such as pranlukast (ONO-1078), a leukotriene receptor antagonist reported to impair GB spread along white matter tracts in certain preclinical models^49^.

**Table 1:** Table summarising the inhibitors used in the drug screen, including the compound name, pharmacological target, IC50, provider, and catalogue number.

Compound name	Pharmacological Target	IC50 in nM	Company	Catalogue number
API-2	Protein kinase B (PKB/Akt)	50	Tocris	2151
PF 573228	Focal Adhesion Kinase (FAK)	4	Tocris	3239
ONO 1078 (Pranlukast)	Cysteinyl leukotriene receptor 1 (CysLT1)	4	Tocris	3026/50
Blebbistatin	Myosin II ATPase	2000	Tocris	1760
Latrunculin B	actin polymerization	60	Tocris	3974
Y-27632	Rho-associated protein kinase (ROCK)	140	Tocris	1254
PD 173074	Fibroblast Growth Factor Receptor (FGFR)	5	Tocris	3044
HPI 1	Sonic Hedgehog	150	Tocris	3839
IWP 2	Wnt	27	Tocris	3533
ALW II-41–27 (Compound 7)	Ephrin receptor EphA2	12	Selleck Chem	S6515
NVP-BHG712 S2202	Ephrin receptor EphB4	25	Selleck Chem	S2202
Ehp-inhibitor-1	Ephrin receptors (EphB2, EphB4 and related Eph kinases)	10	Selleck Chem	S0256
Cilengitide (EMD121974)	integrins αvβ3 and αvβ5	4.1	Tocris	5870
Motixafortide (BL-8040)	stromal derived factor 1 (SDF-1, CXCL12)	1	Selleck	S9665
GF 109203X	Protein Kinase C	8.4	Tocris	0741
SB 431542	TGF-β receptors	94	Tocris	1614
AC 710	Platelet-Derived Growth Factor Receptor (PDGFR)	1.2	Tocris	5013
Iressa	Epidermal growth factor receptor (EGFR)	23	Tocris	3000

**Fig. 6:**
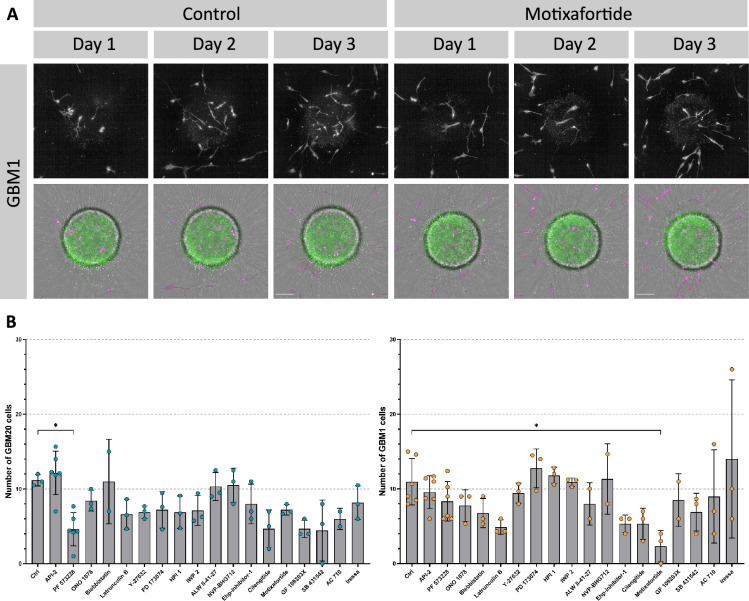
Pilot drug screen on the co-culture of neural spheroids with GBM1 or GBM20 cells. (**A**) Representative maximum projection confocal images acquired on the Operetta CLS showing the co-culture of the neural spheroids with GBM1 cells of Days 1 to 3 in the untreated control and with the inhibitor motixafortide at 1 μM. Scale bar = 100 μm. (**B**) Quantification of GBM20 and GBM1 cellular infiltration at Day 3. Data are baseline-corrected cell numbers and are presented as individual values with mean ± SD from three to six independent experiments (n = 3-6). Statistical significance was assessed using the Kruskal-Wallis test followed by Dunn’s post hoc multiple comparisons test (*, *p* < 0.05).

To validate the platform for pharmacological screening and to probe the mechanism of invasion, we co-cultured labelled GB and NS17 cells with neural spheroids (Day 0). The representative images (Fig. 6A) show progressive GBM1 cell accumulation within and around the spheroid in vehicle-treated controls, and similarly, following motixafortide treatment. Consistent with our previous observations (Fig. 5B), both GBM1 and GBM20 cells progressively infiltrated the neural spheroid over three days. By Day 3, inhibitor-specific effects revealed striking differential sensitivities between the cell lines. The GBM20 infiltration was significantly attenuated by the inhibitor targeting FAK (PF 573228, *p* = 0.0375) (Fig. 6B). Conversely, GBM1 infiltration was refractory to this compound, showing significant reduction only to the CXCR4 antagonist (motixafortide, *p* = 0.0323). These data confirm the platform’s utility in identifying differential therapeutic responses and highlight cell-specific invasion mechanisms.

The observed responses to the inhibitors were specific to the GB lines, as treatment did not significantly alter the infiltration of non-cancerous NS17 cells, which remained consistently low across all conditions and comparable to vehicle-treated controls (See Figure S1B). We further confirmed that this reduction in infiltration had a specific anti-migratory effect and not a consequence of general cytotoxicity. GBM1 cells treated with inhibitors maintained a normal, non-apoptotic morphology over three days, comparable to vehicle controls in both cellular roundness (e.g., 0.38 ± 0.05 for control versus 0.33 ± 0.02 for motixafortide-treated cells at Day 1) and width-to-length ratio (0.29 ± 0.03 for control versus 0.23 ± 0.01 for motixafortide-treated cells at Day 1) (see Figure S1C). These data indicate that the pharmacological inhibition of invasion is not an artefact of drug-induced cell death.

### GBM20 and GBM1 exhibit distinct transcriptomic signatures that align with differential drug sensitivities

To elucidate the transcriptional programs underpinning the distinct infiltrative phenotypes and differential drug sensitivities observed between GBM20 and GBM1, we performed bulk RNA-sequencing. Differential expression analysis was used to identify intrinsic molecular signatures that could account for the functional heterogeneity and variable drug responses between these patient-derived lines.

**Fig. 7:**
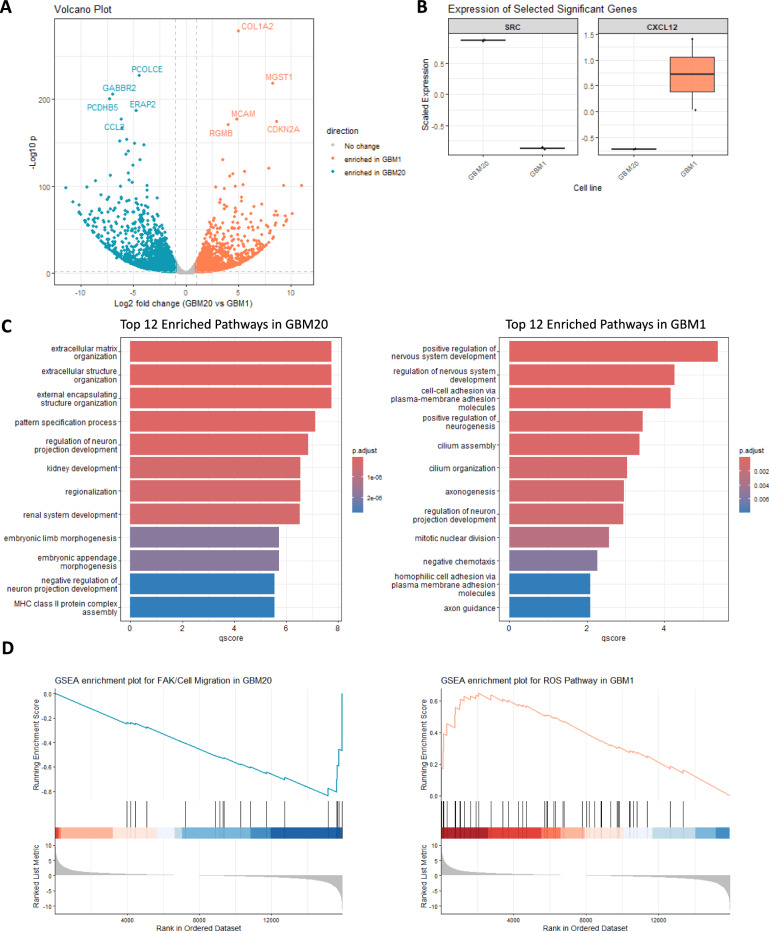
Differential gene expressions between GBM20 and GBM1 cell lines. (**A**) Volcano plot showing the differentially expressed genes between GBM20 (blue) and GBM1 (orange). Genes with an adjusted *p*-value < 0.05 and |log_2_ fold change|> 1 were considered significant. (**B**) Box plots showing the differential expression of the *SRC* and *CXCL12* genes between GBM20 (blue) and GBM1 (orange). (**C**) Bar plots of GO biological process enrichment for significantly upregulated genes in GBM20 and GBM1. The q-score represents enrichment strength (− log₁₀ adjusted *p*-value). Colour intensity corresponds to the adjusted p-value (FDR-corrected), with red indicating higher significance and blue lower significance. (**D**) GSEA enrichment scores of relevant pathways in GBM20 (blue) and GBM1 (orange).

The two GB lines exhibited distinct transcriptomic profiles (Fig. 7A). GBM1 exhibited elevated expression of genes associated with a mesenchymal and pro-migratory phenotype, including the collagen gene *COL1A2* and the motility promoter *MCAM*, while increased *CDKN2A* expression may indicate stress-induced cell cycle arrest or senescence accompanying this phenotypic shift. Conversely, GBM20’s profile was enriched for genes involved in cell-cell interaction (*PCDHB5*), extracellular matrix remodelling (*PCOLCE*), and immune microenvironment signalling (*CCL2, ERAP2*). These divergent transcriptional profiles underpin, in part, the heterogeneous invasive behaviours observed in our assays.

Gene Ontology (GO) enrichment analysis of significantly dysregulated genes (adjusted *p* < 0.05, |log_2_ FC|> 1) further highlighted these differences (Fig. 7C). GBM1 was enriched for neurodevelopmental programs such as “positive regulation of nervous system development” and “axonogenesis,” as well as cell adhesion and guidance processes (“cell–cell adhesion via plasma membrane adhesion molecules” and “axon guidance”) and cilium-associated programs (“cilium assembly” and “cilium organisation”). In contrast, GBM20 showed enrichment for extracellular matrix (ECM) organisation pathways (“ECM structural organisation” and “external encapsulating structure organisation”) and genes involved in neurite outgrowth and synapse formation (“pattern specification process” and “regulation of neuron projection development”).

Gene Set Enrichment Analysis (GSEA) using MSigDB gene sets (GO, KEGG, Reactome, and Hallmark) was performed to determine whether the observed transcriptional differences could account for the differential drug sensitivities. The sensitivity of GBM1 to the CXCR4 antagonist Motixafortide is consistent with its transcriptomic profile, showing enrichment of *CXCL12* expression (Fig. 7B), a chemokine that activates CXCR4 signalling and downstream ROS production. This is reflected by the elevated ROS Pathway signature, linking chemotactic signalling to redox regulation (Fig. 7D). In contrast, the sensitivity of GBM20 to the FAK inhibitor PF 573228 corresponds with enrichment of *SRC* (Fig. 7B), a key downstream mediator of the cell migration/FAK pathway involved in focal adhesion turnover and cytoskeletal remodelling required for motility (Fig. 7D). Together, these differential expression and GSEA results provide a direct molecular rationale for the distinct pharmacological vulnerabilities observed in the patient-derived glioblastoma cell lines.

## Discussion

We present a physiologically relevant *in vitro* co-culture model combining patient-derived GB cells with human iPSC-derived neural spheroids. These spheroids exhibit highly consistent size and morphology, forming a dense core of neuronal cell bodies with radially extending axons, thereby providing a reproducible and scalable platform for assessing GB cell infiltration. Using high-content imaging, we established automated pipelines to quantify axon engagement, directional migration in live assays, and spheroid infiltration in endpoint assays. This iPSC-derived neural spheroid platform offers distinct advantages over existing models. While traditional 2D migration assays are high-throughput, they lack the complex 3D cytoarchitecture and cell-cell interactions of the human brain microenvironment^50,51^. Conversely, *in vivo* animal models are challenged by their translational relevance to human-specific GB phenotypes^52^. Our platform bridges this gap, offering a scalable, human-relevant 3D co-culture system that successfully models key aspects of GB invasion. Moreover, *in vitro* models have failed to recapitulate complex migratory behaviours, such as contact guidance along white matter tracts, a primary mechanism of GB infiltration^53^. Our axon-focused platform, for the first time, quantifies this behaviour through directionality analysis and infiltration counts to study the tumour-brain interactions.

Our results demonstrated that while both GB lines and a non-tumourigenic neural stem cell line (NS17) migrated toward neural spheroids, only GB lines infiltrated them. This suggests that while neural-derived cues attract all lines, only GB cells possess the mechanism for infiltration. We report that GBM1 infiltrated more readily than GBM20 over five days, acknowledging that this metric combines both migration and proliferation, making it difficult to dissect the two. In contrast, prior work showed greater aggressiveness with patient-derived GBM20 spheroids. Irving (2020) showed GBM20 spheroids spontaneously fused and infiltrated human cerebral organoids faster than GBM1 spheroids^54^. These differences likely reflect treatment-induced phenotypic shifts, as GBM20 is derived from a recurrent tumour exposed to radiotherapy and chemotherapy^55^. Notably, recurrent GB cells adopt less proliferative, pre-oligodendrocyte-like states in response to damage^49^. Accordingly, the reduced infiltration of GBM20 possibly reflects a combination of both reduced migratory potential and a less proliferative state.

A focused drug screen elucidated line-specific sensitivities, with specific compounds reducing GBM20 and GBM1 infiltration, establishing proof-of-principle for our platform as a tool to identify anti-infiltration compounds. This screen uncovered distinct molecular dependencies: GBM20 was selectively sensitive to inhibition of FAK (PF 573228), while GBM1 responded to CXCR4 blockade (motixafortide), a pathway previously implicated in GB immunotherapy^48^. Furthermore, qualitative and quantitative analysis of the cellular morphology of drug-treated GBM1 cells supported that any reductions in infiltration were not due to cytotoxicity or cell death but specific anti-invasive effects.

Transcriptomic analyses mirrored these pharmacological sensitivities: GBM20 showed upregulation of the *SRC* gene and the FAK/Cell Migration pathway, consistent with responsiveness to PF 573228. This aligns with previous studies testing PF 573228 against well-established GB cell lines, including T98G and U-118^44^. Conversely, elevated *CXCL12* expression and enhanced ROS pathway activity in GBM1 underlie its heightened responsiveness to motixafortide, resulting in a more pronounced reduction in cellular infiltration. Together, these findings underscore the utility of integrating transcriptomics with functional migration assays to uncover subtype-specific invasion programs and therapeutic vulnerabilities. Furthermore, our approach complements recent advances using machine learning and high-throughput drug screening^56^. Our results advocate for the heterogeneity of GB and establish patient-derived co-culture systems as a scalable platform for necessary functional screening. Importantly, patient-specific analysis allows direct correlation of molecular signatures with functional phenotypes and drug responses. By modelling tumour-brain interactions in a human-relevant context, our platform contributes to the growing adoption of NC3R- and NAM-aligned approaches that reduce reliance on animal models while enhancing translational relevance^57,58^.

### Limitations and future directions

We acknowledge the limitations of this work. This pilot study included only two GB lines and a single control, limiting the generalisability of our findings. Expanding the model to a larger and more diverse panel of patient-derived lines will enhance its predictive utility for therapeutic development and enable more robust, patient-specific analyses of infiltration and drug response.

Observation of the migratory cell behaviour with live imaging prompted us to resort to ptycography (via the Phasefocus Livecyte device) to quantify and perform a directionality analysis. This showed a significant biological effect (*p* < 0.0001), based on a large number of cells, albeit from a single experimental replicate. While the Rayleigh and Chi-Squared analyses confirm this effect is not due to random chance, further work is needed to substantiate these results. Additionally, as discussed, our current infiltration metric does not distinguish between migration and proliferation, a challenge that could also be addressed in future work with a full seeding titration and a retrospective quantification of inside vs. outside ratios for all lines. Technical refinements could further enhance the physiological relevance of the platform. While phase-contrast imaging enabled live tracking of GB migration and pseudopodia formation^59–61^, it was suboptimal for visualising infiltration within dense neural spheroids. Integrating our current high-content, high-throughput pipeline with live-cell imaging on the Operetta CLS will allow quantitative analysis of infiltration dynamics in 3D. Future iterations will also include additional neural cell types, glia, or vascular components to model the tumour microenvironment more comprehensively. Indeed, incorporating iPSC-derived oligodendrocytes would better mimic myelinated tracts, as myelin affects glioma migration^59,62^.

The broader implications of this platform are significant, particularly in translational contexts. By integrating patient-derived GB cells, high-content imaging, and molecular profiling, it provides a scalable, human-specific preclinical tool for identifying subtype-specific vulnerabilities, screening anti-infiltration compounds, and informing personalised treatment strategies. Its high-throughput and reproducible design extends beyond 2D viability assays to assess GB invasion phenotypes in a physiologically relevant context.

### Conclusion

Glioblastoma invasion and migration remain poorly understood despite extensive study of tumour growth. Our work provides a key conceptual and technical advance: a human iPSC-derived neural spheroid co-cultured with patient-derived GB cells. This axon-extending, physiologically relevant platform enables direct investigation of tumour infiltration. We link molecular profiles to functional behaviours and screen a drug panel to reveal patient-specific GB vulnerabilities. By integrating high-content imaging, drug screening, and transcriptomic profiling, we captured distinct, cell-line-dependent, migratory programs and drug sensitivities. Together, we offer a novel, preclinical tool to better understand GB invasion and accelerate the development of personalised therapeutics.

## Methods

### Cell culture

#### GB cells culture

The patient-derived GBM1 and GBM20 cell lines were derived under informed consent according to ethical approvals (LREC 115/ES/0094; courtesy of Dr Heiko Wurdak, University of Leeds)^9,35^. GBM1 and GBM20 cell lines were derived, respectively, from a primary tumour of a 58-year-old female patient and a 50-year-old male patient with a recurrent GB, previously treated with radiotherapy, temozolomide, and IMA950. The cell models maintain the stem cell-like characteristics of GB under adherent culture conditions^9,35,63^.

Cells were cultured 5 μg/mL poly-L-ornithine (PLO) (Sigma P3655-50MG) and 2 μg/mL laminin-coated (Thermofisher 23017015) plates in Neurobasal medium (Invitrogen 21103049) supplemented with 0.5X B27 (Invitrogen 17504044), 0.5X N2 (Invitrogen 17502048), 40 ng/mL epidermal growth factor (EGF) (R&D Systems 236-EG-200), and 40 ng/mL basic-fibroblast growth factor (b-FGF) (Invitrogen PHG0026). Cells were passaged using 1X TrypLE (Gibco 12604013) when approximately 80% confluency was reached, and passages remained between passage numbers 14 to 20. Cell cultures were routinely assessed for mycoplasma, and all were negative for contamination.

#### Neural stem cells

The cells GCGR.NS17ST_A (shortened to NS17) from the Glioma Cellular Genetics Resource (GCGR) was acquired under informed consent according to local ethical approvals (08/S1101/1)^36^ and kindly provided by Dr Steven Pollard (University of Edinburgh). The cells were cultured according to previously published methods in DMEM/HAMS-F12 medium (Sigma D8437) supplemented with 1.5 mg/mL of glucose (Sigma G8644), 100 μM MEM NEAA (Gibco 11140–035), 50 U/mL and 50 mg/mL Penicillin/Streptomycin (Gibco 15140–122), 0.01% BSA (Gibco 15260–037), 100 μM beta-mercaptoethanol (Gibco 31350–010), 0.5X B27, 0.5X N2, EGF to 10 ng/mL, and b-FGF to 10 ng/mL. The cells were passaged using StemPro Accutase Cell Dissociation Reagent (Gibco A1110501) when they reached approximately 80% confluency.

#### Induced glutamatergic neuron cell culture

HPSI1013I-PAMV_1 human iPSCs obtained from the HipSci biobank, where pluripotency characterisation, such as Pluritest, was performed as part of their standard quality control pipelines (www.hipsci.org). The line was derived from skin fibroblasts from a healthy male donor aged 65-69 from a White British ethnicity. HPSI1013I-PAMV_1 cells were transduced with a doxycycline-inducible Neurogenin-2 (iNGN2) transgene following the protocol from the Ward laboratory^34^. The integration of the transgene was achieved by electroporation and TALEN-mediated integration into the AAVS1 safe-harbour locus. iNGN2 PAMV_1 cells were cultured in feeder-free conditions on 4% Vitronectin XF (Stem Cell Technologies 100–0763) coated plates in Essential 8 medium (Gibco A1517001) supplemented with 50 U/mL and 50 mg/mL Penicillin/Streptomycin, respectively. The medium was changed daily, and cells were passaged using TrypLE and resuspended in Essential 8 medium with 10 μM Y-27632 Rho-kinase (ROCK) inhibitor (ENZO Life Sciences ALX-270-333-M005) at approximately 1.5 million cells per well. All cell cultures were routinely screened for mycoplasma and confirmed to be negative for contamination.

#### Transcription factor-mediated differentiated CNS-like glutamatergic neurons

The transcription factor-mediated differentiation protocol was adapted from the Ward laboratory^34^. On Day 0, iNGN2 PAMV_1 cells were dissociated using Accutase at 37°C for 5 min. The cells were resuspended with the Induction Medium—DMEM/F12 Hepes medium (Life Technologies 11330032), supplemented with 0.5X N2, 100 μM MEM NEAA, 2 mM L-Glutamine (Gibco 25030081), Doxycycline (2 μg/ml) (Sigma D9891), and 10 μM Y-27632 ROCKi (1:100), on GFR Matrigel (BD Biosciences 356230) (1:50 in DMEM) at a density of 1.5 million cells per well. Doxycycline will induce the differentiation into Glutamatergic Excitatory Neurons. On Days 1 and 2, nascent neurites began to be evident, and the media was replaced with fresh induction media with Doxycycline. Cells were dissociated with Accutase for freezing in 10% dimethyl sulfoxide (Sigma D2650) in medium or for neural spheroid generation.

#### Generation of neural spheroids

Following dissociation, differentiated cells resuspended in the induction media were seeded at a density of 10,000 cells/well in the 5% Pluronic F127 (Sigma-Aldrich P2443) coated V-bottom plate and centrifuged for 2 min at 200 × g to aggregate the cells at the bottom of the well according to the in-house protocol^64^. The cells were left for 48 h with daily half-medium changes.

#### Maturation and generation of axon bundles

On Day 6, the neural spheroids were transferred to laminin-coated 24-well or 96-well glass-bottom plates (Cellvis P24-0-N and P96-0-N) in Cortical Neuron Culture Medium—BrainPhys neuronal medium (Stemcell Technologies 5790) supplemented with 0.5X B27, 10 ng/mL Brain-Derived Neurotrophic Factor (BDNF) (PeproTech 450–02), 10 ng/mL Neurotrophin-3 (NT-3) (PeproTech 450–03), and 1 μg/mL Laminin. The cells were checked daily for the presence of cell debris and morphological changes with bi-weekly half-medium changes.

#### Co-culture of GB or NS17 cells with neural spheroids

On Day 11, 1X BioTracker 488 Green Nuclear Dye (Sigma-Aldrich SCT120) was added to the cell culture medium to stain the neural spheroids for 20 min at 37°C and washed twice. GB cells were labelled with 1X BioTracker 655 Red Cytoplasmic Membrane Dye (Sigma-Aldrich SCT108) in suspension for 20 min at 37°C and washed three times by centrifugation. The GB or NS17 cells were seeded on top of the neural spheroids at a density of 5,000 cells/well in a 24-well plate or 840 cells/well in a 96-well plate with a 50/50 medium.

### Immunofluorescence staining and imaging

#### Immunostaining

Neural spheroids were fixed at 4% PFA/PBS at RT for 20 min and permeabilised with 3% BSA/0.1% Triton-X-100/PBS for 1 h at RT. Cells were incubated overnight at 4°C in the dark with the primary mouse axonal antibody βIII-tubulin (Tubb3) (R&D Systems MAB1195) diluted in 3% BSA/0.1% Triton-X-100/PBS (1/1000). The next day, primary antibodies were rinsed away with 3% BSA/PBS. The cells were incubated with the secondary anti-mouse antibody conjugated to AF 488 (Invitrogen A-11029), diluted in 3% BSA/0.1% Triton-X-100/PBS (1:500) at RT for 45 min and rinsed with PBS. To stain the nucleus, the cells are incubated with 10 μM Hoechst (Invitrogen 62248) for 10 min at RT and rinsed with PBS. Finally, the cells were incubated with 80% glycerol as a clearing agent for 1 h at RT and rinsed with PBS before being placed in fresh PBS at 4°C until imaging.

#### Confocal microscopy

Confocal images were acquired using a Leica TCS SP8 Confocal laser scanning microscope, using a 10 × dry objective, and viewed with the Leica software. Each fluorochrome was excited with the corresponding laser line (DAPI, UV (355 nm); AF488, Green (530 nm); AF594, Red (639 nm) laser line). Immunofluorescence data were analysed in Fiji open-source software (2.0.0-rc-64/1.51s version). Images from different fields were tiled and stitched, and the maximum projection was obtained using the Z-stack.

### Live-cell imaging assay

#### Live-cell imaging of non-labelled cells

Live-cell imaging of non-labelled cells on laminin-coated glass plates and on axons was performed on the Livecyte quantitative phase imager (Phasefocus), which uses quantitative phase imaging with an accurate tracking apparatus. ROIs (10 mm × 25 mm) on the left and the top of the neural spheroids were selected, and images were acquired every 15 min for 24 h.

#### Live-cell imaging assay analysis

Images were analysed using the built-in Analysis Software with the Motility Assay on the Livecyte quantitative phase imager (Phasefocus). Using the built-in applications, the cells were identified and tracked, and the tracking properties were calculated to generate cell migration parameters. Directionality results were analysed in a polar histogram and with the Horizontal Preference Index (HPI) using R Studio (version 2025.05.1) and the GraphPad Prism software (version 10.6).

### Endpoint imaging assay

#### Endpoint imaging assay of labelled cells

Co-cultures of patient-derived GB cells and human iPSC-derived cortical-like neural spheroids were imaged every 24 h at Days 0, 1, 2, and 3 using the Operetta CLS High Content Analysis (PerkinElmer) with the brightfield, 488 nm, and 655 nm channels. The Operetta is a confocal microscope that uses a spinning disk and a built-in Harmony High-Content Imaging and Analysis Software. The PreciScan Intelligent Acquisition plug-in for Harmony software was used to locate the neural spheroid within the well. The plug-in allows the accurate targeting of the region of interest (ROI) whilst reducing acquisition and analysis times. Once the PreciScan was performed and the region of the neural spheroid was located, a Z-stack of 10 images over 20 μm (distance 2 μm) was acquired within the spheroid depth.

#### Endpoint assay analysis using harmony

Images were analysed using the built-in Harmony Software. The maximum projection of the Z-stack was taken, and the neural spheroid stained with the BioTracker 488 was identified as an ROI using the green channel. The GB cells stained with the BioTracker 655 in the ROI were identified and segmented using the far-red channel (Segmentation Method C in Harmony Software).

All experiments were performed in technical triplicate and were independently repeated at least three times to perform statistical analysis. All statistical analyses were performed using GraphPad Prism software (version 10.6). Results were baseline-corrected to Day 0 and are represented as means with standard deviation (SD). Two-way repeated-measures ANOVA with Geisser–Greenhouse correction, followed by Tukey’s multiple comparisons, were performed on log_10_(Y + 1) transformed data to address equal variance and to evaluate cell line by time interaction. A p-value below 0.05 was considered significant and was indicated with an asterisk: *, *p* ≤ 0.05; **, *p* ≤ 0.005; ***, *p* ≤ 0.005. The number of independent experiments is indicated in the figure legend as n.

#### Inhibitor screen using endpoint assay

Inhibitors were added to the co-culture at a final concentration of 1 μM. Following a 6 h antagonist treatment, images were acquired using the Operetta CLS Imaging System over three days. Images were analysed as described above for the endpoint assay. The non-parametric Kruskal–Wallis test followed by Dunn’s post hoc multiple comparisons test was performed to evaluate the inhibitor effect at Day 3 (*, *p* < 0.05).

### RNA sequencing

The GBM1 and GBM20 cell lines were prepared for RNA sequencing. They were rinsed once with PBS, detached with TrypLE, and snap-frozen in pellets of 10^6^ cells. The cells were submitted to Eurofins Genomics, Germany, under Project ID: NG-29040. Raw sequencing data were pre-processed using the fastp software to generate clean data. This involves checking the quality of the raw sequencing filtering for high-quality reads to remove poor-quality bases (below Phred Quality 20)^65^.

High-quality sequence reads were aligned using the STAR (Spliced Transcripts Alignment to a Reference) to the reference genome UCSC Homo sapiens version hg38^66^. Gene-wise quantification was achieved to inspect the transcriptome alignments using the RSEM tool^67^. For the differential gene expression between cell lines, genes with fewer than 10 average reads were removed. Using the R/Bioconductor DESeq2 package, the abundance counts of each gene were then used to perform differential gene expression^68^. Eurofins Genomics provided this pre-processing.

### RNA-sequencing data analysis

Further analysis was performed on R Studio using the ggplot2 package to generate a volcano plot and boxplots of differential gene expression. Sample-wise comparison values (log_2_ fold change and p-value) provided by Eurofins Genomics were used. Gene Ontology (GO) enrichment and Gene Set Enrichment Analysis (GSEA) were performed using the clusterProfiler and fgsea packages to identify biological processes and pathways associated with differential expression. Significantly enriched terms were visualised using enrichment plots.

## Supplementary Information

Supplementary Video 1. GBM1 with axons

Supplementary Video 2. GBM1 without axons

Supplementary Video 3. GBM20 with axons

Supplementary Video 4. GBM20 without axons

Supplementary Video 5. NS17 with axons

Supplementary Video 6. NS17 without axons

Supplementary Information 1.

Supplementary Information 2.

## Data Availability

Processed data have been deposited in the Gene Expression Omnibus (GEO) under accession number GSE308010. Raw sequencing data (BAM files) are available in the Sequence Read Archive (SRA) under BioProject accession number PRJNA1327912.
