# Supplementary figures and images for: A human iPSC-based neural spheroid platform for modelling glioblastoma infiltration using high-content imaging
Source: Sci Rep. 2025 Dec 13;16:1223. doi: 10.1038/s41598-025-30914-5 (PMC12789424; doi:10.1038/s41598-025-30914-5)

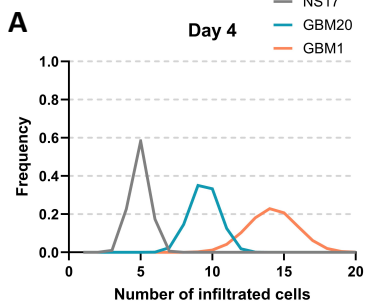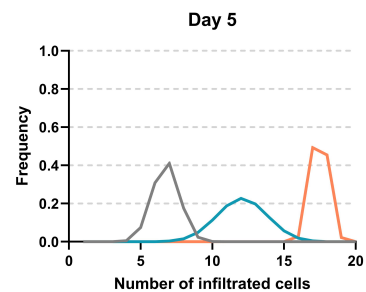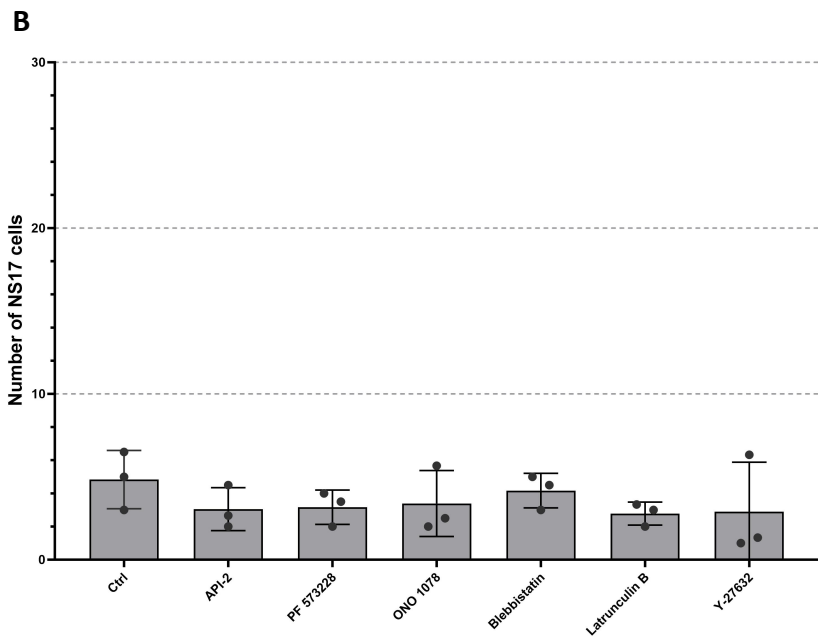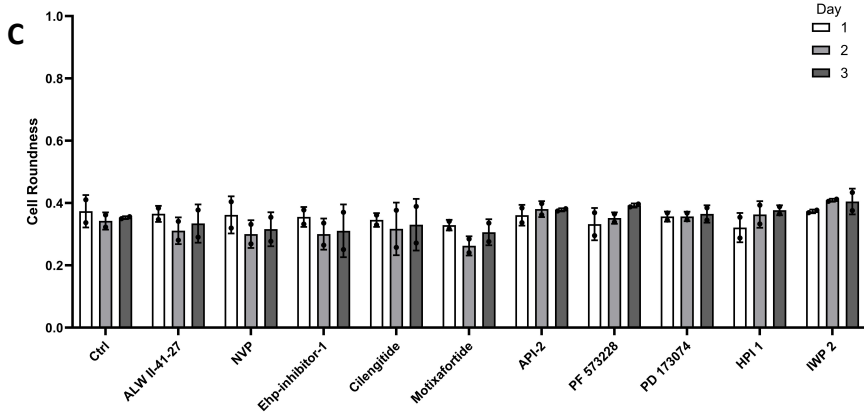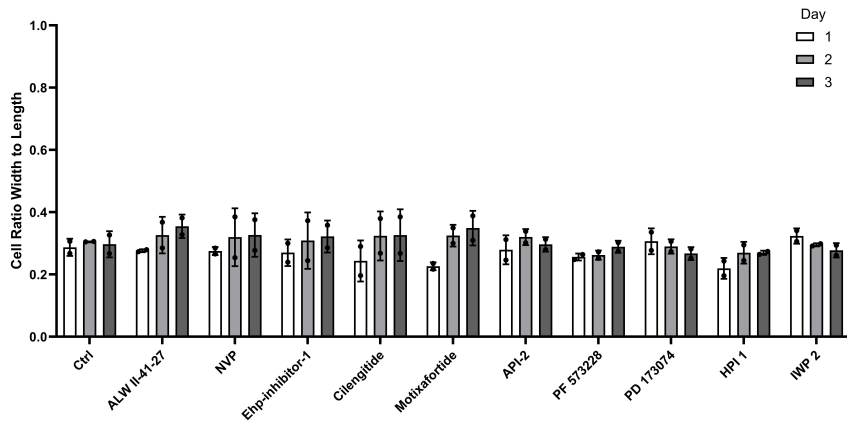

Supplement: Supplementary file 7 — Supplementary Information 1. [file 41598_2025_30914_MOESM7_ESM.pdf]
