# Supplementary material for: A human iPSC-based neural spheroid platform for modelling glioblastoma infiltration using high-content imaging
Source: Sci Rep. 2025 Dec 13;16:1223. doi: 10.1038/s41598-025-30914-5 (PMC12789424; doi:10.1038/s41598-025-30914-5)
Supplement: Supplementary file 8 — Supplementary Information 2. [file 41598_2025_30914_MOESM8_ESM.docx]

A human iPSC-based neural spheroid platform for modelling glioblastoma infiltration using high-content imaging.

**Victoria S. K. Tsang^1,7^*, Federica Riccio^1^, Aimee S. Wilson^4^, Hannah Nudds^1,8^, Jason D. Coombes^1,9^, Heiko Wurdak^6^, Harry J. C. J. Bulstrode^4,5^, Ivo Lieberam^1,3^ and Davide Danovi^1,2,4,10^**

^1^Centre for Gene Therapy and Regenerative Medicine, King’s College London, London, United Kingdom

^2^Department of Basic & Clinical Neuroscience Institute of Psychiatry, Psychology & Neuroscience King’s College London

^3^Centre for Developmental Neurobiology and MRC Centre for Neurodevelopmental Disorders, King’s College London, London, United Kingdom

^4^Wellcome MRC Cambridge Stem Cell Institute, University of Cambridge, Cambridge, United Kingdom

^5^Department of Neurosurgery, Addenbrooke's Hospital, Cambridge, United Kingdom

^6^School of Medicine, University of Leeds, Leeds, United Kingdom

^7^present address: Translational Medicine and Therapeutics, Queen Mary University of London, London, United Kingdom

^8^present address: Department of Clinical and Movement Neurosciences, University College London, London, United Kingdom

^9^present address: Saint Louis University, St Louis, United States

^10^Migration Biotherapeutics, Cardiff, United Kingdom

*Requests for further information and resources should be directed to and will be fulfilled by the lead contact ([v.tsang@qmul.ac.uk](mailto:v.tsang@qmul.ac.uk)).

# Supplementary figures

Figure S1.

1. Normal distribution curves for NS17, GBM20, and GBM1 infiltration calculated from the means and standard deviation at Days 4 and 5 (Z-factor = 0.218240948 and 0.574686702, respectively).
2. Quantification of NS17 infiltration of the neural spheroid at Day 3. Data are baseline-corrected cell numbers and are presented as individual values with mean ± SD from three independent experiments (n = 3).
3. Quantification of GBM1 cell morphology to assess potential cytotoxic effects of drug treatment. Roundness and width-to-length ratio were measured as indicators of cell health. number of independent experiments (n) = 2.

Supplementary videos. Neural spheroids co-cultured with NS17, GBM20, and GBM1 cells imaged for 24h.
